# Live virus neutralization testing in convalescent patients and subjects vaccinated against 19A, 20B, 20I/501Y.V1 and 20H/501Y.V2 isolates of SARS-CoV-2

**DOI:** 10.1080/22221751.2021.1945423

**Published:** 2021-08-01

**Authors:** Carla Saade, Claudia Gonzalez, Antonin Bal, Martine Valette, Kahina Saker, Bruno Lina, Laurence Josset, Mary-Anne Trabaud, Guillaume Thiery, Elisabeth Botelho-Nevers, Stéphane Paul, Paul Verhoeven, Thomas Bourlet, Sylvie Pillet, Florence Morfin, Sophie Trouillet-Assant, Bruno Pozzetto, on behalf of COVID-SER study group

**Affiliations:** a Laboratoire de Virologie, Institut des Agents Infectieux, Laboratoire associé au Centre National de Référence des virus des infections respiratoires, Hospices Civils de Lyon, Lyon, France; b Virpath, CIRI, INSERM U1111, CNRS UMR5308, ENS Lyon, Université Claude Bernard Lyon 1, Villeurbanne, France; c Service de médecine intensive réanimation, Centre Hospitalier Universitaire de Saint-Etienne, Saint-Etienne, France; d CIRI, équipe GIMAP, Université de Lyon, Université de Saint-Etienne, INSERM U1111, CNRS UMR5308, ENS de Lyon, UCBL1, Saint-Etienne, France; e Service d'Infectiologie, Centre Hospitalier Universitaire de Saint-Etienne, 42055 Saint-Etienne, France; f Département d’immunologie, Centre Hospitalier Universitaire de Saint-Etienne, Saint-Etienne, France; g Département des agents infectieux et d’hygiène, Centre Hospitalier Universitaire de Saint-Etienne, Saint-Etienne, France

**Keywords:** SARS-CoV-2, humoral response, variant of concern, live virus neutralization test, 20B, 20I/501Y.V1, 20H/501Y.V2

## Abstract

SARS-CoV-2 mutations appeared recently and can lead to conformational changes in the spike protein and probably induce modifications in antigenicity. We assessed the neutralizing capacity of antibodies to prevent cell infection, using a live virus neutralization test with different strains [19A (initial one), 20B (B.1.1.241 lineage), 20I/501Y.V1 (B.1.1.7 lineage), and 20H/501Y.V2 (B.1.351 lineage)] in serum samples collected from different populations: two-dose vaccinated COVID-19-naive healthcare workers (HCWs; Pfizer-BioNTech BNT161b2), 6-months post mild COVID-19 HCWs, and critical COVID-19 patients. No significant difference was observed between the 20B and 19A isolates for HCWs with mild COVID-19 and critical patients. However, a significant decrease in neutralization ability was found for 20I/501Y.V1 in comparison with 19A isolate for critical patients and HCWs 6-months post infection. Concerning 20H/501Y.V2, all populations had a significant reduction in neutralizing antibody titers in comparison with the 19A isolate. Interestingly, a significant difference in neutralization capacity was observed for vaccinated HCWs between the two variants but not in the convalescent groups.

Several SARS-CoV-2 variants of concern (VOC) with mutations impacting notably the spike (S) protein have been detected recently [1]. These mutations can lead to conformational changes and probably induce modifications in antigenicity. Serological studies based on SARS-CoV-2 pseudotyped or chimeric viruses have been performed to measure the neutralization activity of serum specimens of convalescent patients or subjects immunized by SARS-CoV-2 vaccines. Although these tests are easier to conduct, their ability to predict neutralizing activity against authentic clinical viral isolates needs to be evaluated [2].

Herein we compared the ability of neutralizing antibodies (NAb) directed against SARS-CoV-2 to prevent cell infection in different populations, using a live Virus Neutralization Test (VNT) against SARS-CoV-2 isolates belonging to various clades: 19A, 20B (B.1.1.241 lineage), 20I/501Y.V1 (B.1.1.7 lineage) and 20H/501Y.V2 (B.1.351 lineage). Each SARS-CoV-2 isolate used in this study was sequenced and confirmed to harbour the characteristic mutations of its viral lineage. VNT was performed as previously reported using an observed viral load of 100 to 500 50% Tissue Culture Infectious Doses (TCID_50_) for each isolate [3,4].

Serum specimens were collected from two-dose vaccinated COVID-19 naïve healthcare workers (HCWs; *n* = 30) between two and four weeks after the administration of the Pfizer-BioNTech BNT162b2 vaccine (group 1), a subgroup of HCWs exhibiting significant NAb against 19A (50% plaque reduction neutralization test (PRNT_50_) ranging from 20 to 240) 6 months after mild COVID-19 (*n* = 29; group 2) [5], and critical COVID-19 patients sampled within one month after symptom onset (median [interquartile range, IQR]: 28 [22.5–33.5] days; *n* = 25; group 3). All COVID-19 patients from groups 2 and 3 had been infected during the first wave of COVID-19 that occurred in France in March–April of 2020. Written informed consent was obtained from all HCWs; ethics approval was obtained from the national review board for biomedical research in April 2020 (Comité de Protection des Personnes Sud Méditerranée I, Marseille, France; ID RCB 2020-A00932-37), and the study was registered on ClinicalTrials.gov (NCT04341142). Concerning critical patients, this study was approved by the ethics committee of the university hospital of Saint-Etienne (reference number IRBN512020/CHUSTE).

No significant difference in median NAb titres was observed on a subset of serum specimens taken from the three populations between the 19A isolate taken as reference and the 20B isolate that circulated during the second pandemic wave at the end of 2020 in Europe (Figure 1(A)). Even if this result was expected, it had not been reported before; it indicates that the S477N mutation has no impact on the ability of NAbs to confer protection.

**Figure 1.**
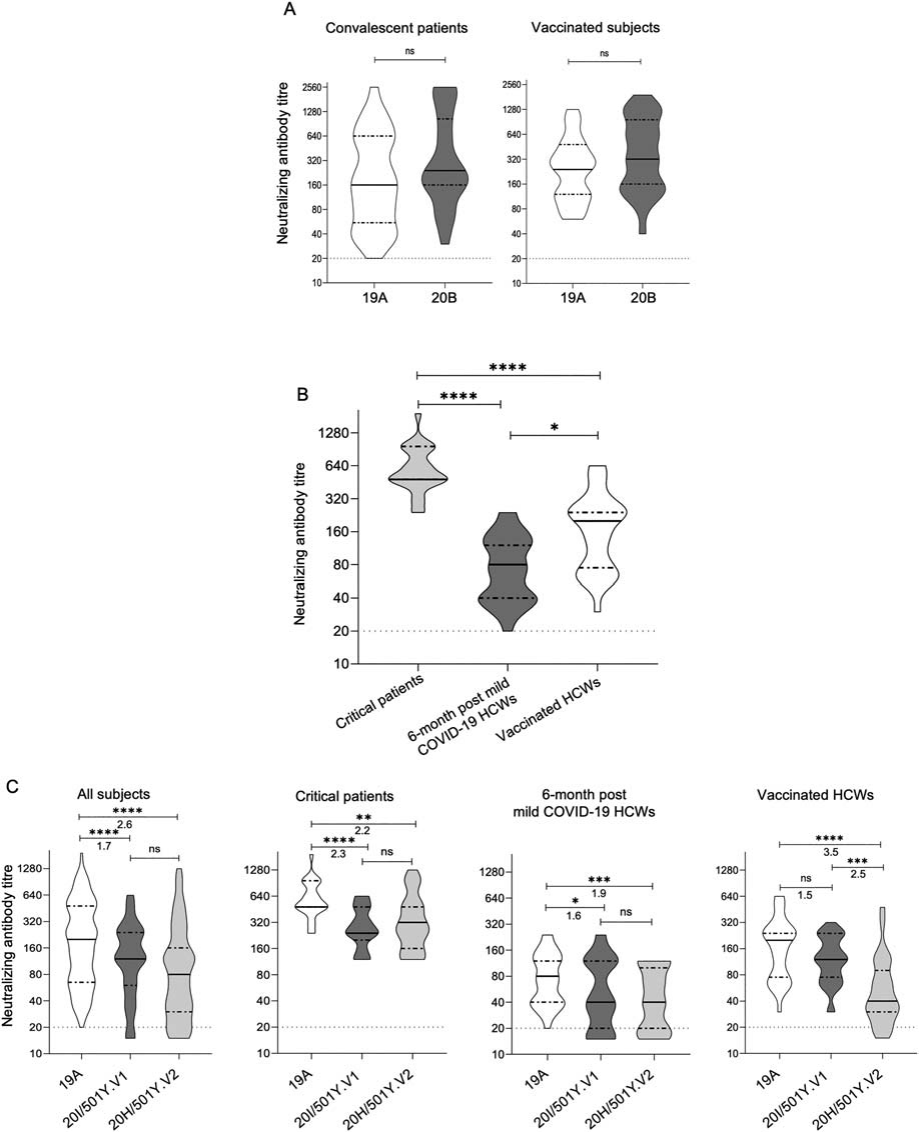
Neutralization of living isolates of SARS-CoV-2 by convalescent sera from critical or mild patients with COVID-19 and by vaccine-elicited sera from subjects having received two doses of the BNT162b2 vaccine. Each serum was tested in duplicate and the mean NAb was used for the analysis. Titres were transformed into log2 values for the calculation of mean NAb titres (a value of 0.5 was attributed by convention to negative samples). Dotted lines represent the detection threshold of 50% plaque reduction neutralization test (PRNT50) ≥20 for neutralizing antibody (NAb) titres; full lines represent median NAb titres and dashdotted lines represent the 25% and 75% quartiles. (A) Violin plots presenting the neutralization of 2 SARS-CoV-2 isolates belonging to the 19A and 20B clades by serum specimens obtained from critical patients (*n* = 27), 6-month post mild COVID-19 healthcare workers (HCWs) (*n* = 19) and vaccinated subjects (*n* = 15); the Wilcoxon matched-pair signed rank test was used for comparisons. (B) Violin plots presenting the neutralization of a SARS-CoV-2 isolate belonging to the 19A clade by serum specimens obtained from vaccinated subjects (*n* = 30), 6-month post mild COVID-19 HCWs (*n* = 29) and critical patients (*n* = 25); the Kruskal-Wallis test followed by Dunn’s multiple comparison test was used for comparisons. (C) Violin plots presenting the neutralization of 3 SARS-CoV-2 isolates belonging to the 19A, 20I/501Y.V1 and 20H/501Y.V2 clades by the same serum specimens than in (B); the Friedman test followed by the Dunn’s multiple comparison test was used for comparisons. For each VOC, the fold reduction in NAb titres compared to 19A is shown under the statics bar. *: *p*-value < 0.05; **: *p*-value < 0.01; ***: *p*-value < 0.001; ****: *p*-value < 0.0001; ns: non-significant.

As previously reported between mild and severe patients [3], the three populations exhibited significantly different median levels of NAb for 19A isolate (Figure 1(B)); the same picture was observed for the two VOC (data not shown).

By comparison to the 19A isolate, the median [IQR] fold reduction in NAb titres was 1.5 [1.2–1.7] and 3.5 [2.7–4.3] in group 1, 1.6 [1.3–1.8] and 1.9 [1.5–2.3] in group 2, and 2.3 [1.8–2.8] and 2.2 [1.5–2.8] in group 3, for the 20I/501Y.V1 and 20H/501Y.V2 VOC respectively (Figure 1(C)). Interestingly, the difference in median NAb titres was highly significant between the two variants (*p* < 0.001) for vaccinated subjects (group 1) whereas it was not significant for the two other groups corresponding to naturally infected patients (Figure 1(C)).

As a whole, critical patients exhibited a strong neutralizing response against all the tested strains; despite a slight reduction in NAb titres for both variants by comparison to the wild-type strain, no neutralization escape occurred against the two VOC due to the high titres of NAb. This population of subjects has not been tested in previous studies involving the neutralization escape to VOC strains; additional data are needed to confirm this reassuring observation.

The 6-month neutralizing response of HCWs with mild COVID-19 was slightly reduced towards both variants by comparison to the wild type strain. By contrast to that reported herein, a study performed with pseudotype viruses on the same category of patients (6-month mild COVID-19 HCWs) found that the neutralizing response was much lower against the 20H/501Y.V2 variant than against the 20I/501Y.V1 one [6], which would suggest an increased risk of neutralization escape with the former strain.

Another striking finding of the present study is the reduced neutralizing response observed towards the 20H/501Y.V2 variant in fully immunized subjects with the BNT162b2 vaccine by comparison to the wild type and 20I/501Y.V1 variant. These results are in accordance with that observed for the same vaccine in numerous studies using pseudotype viruses [6–9] or authentic variant strains [10–13]. The neutralization escape of the 20H/501Y.V2 variant in subjects having received either the BNT162b2 vaccine [11–14] or the Oxford-AstraZeneca AZD1222 vaccine [14] was shown to be mainly mediated by the synergistic effect of mutations K417N, E484K and N501Y in the receptor binding domain. This effect was in part corrected by a hyper-immunization like that observed in naturally infected people whose immunity was boosted by an additional dose of vaccine [15].

Although the present study was performed using a live VNT, it focused only on the humoral response and other experiments are needed to assess the overall immune process including T-cell immune response. Another limitation of the study regards the huge differences between the three tested populations in terms of time post-infection or immunization, which restrains the interpretation of the differences in results between the three groups.

In conclusion, the relative good conservation of the neutralizing activity of sera from the three populations tested in this study against the two variants 20I/501Y.V1 and 20H/501Y.V2 is encouraging towards a putative reinfection by these strains. Long-term monitoring of the NAb response together with that of the specific cellular response will be needed to confirm these favourable findings.

## Data Availability

GISAID accession numbers: EPI_ISL_1707038; EPI_ISL_1707039; EPI_ISL_1707040; EPI_ISL_768828.
